# Breast Milk: A Meal Worth Having

**DOI:** 10.3389/fnut.2021.800927

**Published:** 2022-01-26

**Authors:** Anoud Duale, Parul Singh, Souhaila Al Khodor

**Affiliations:** ^1^Division of Maternal and Child Health, Department of Translational Medicine, Research Branch, Sidra Medicine, Doha, Qatar; ^2^College of Health and Life Sciences, Hamad Bin Khalifa University, Ar-Rayyan, Qatar

**Keywords:** breastfeeding, microbiota, delivery, chronic diseases, immune system

## Abstract

A mother is gifted with breast milk, the natural source of nutrition for her infant. In addition to the wealth of macro and micro-nutrients, human milk also contains many microorganisms, few of which originate from the mother, while others are acquired from the mouth of the infant and the surroundings. Among these microbes, the most commonly residing bacteria are *Staphylococci, Streptococci, Lactobacilli* and *Bifidobacteria*. These microorganisms initiate and help the development of the milk microbiota as well as the microbiota of the gastrointestinal tract in infants, and contribute to developing immune regulatory factors such as cytokines, growth factors, lactoferrin among others. These factors play an important role in reducing the risk of developing chronic diseases like type 2 diabetes, asthma and others later in life. In this review, we will summarize the known benefits of breastfeeding and highlight the role of the breast milk microbiota and its cross-talk with the immune system in breastfed babies during the early years of life.

## Introduction

Breast milk (BM) is the normative source of nutrition for infants in the first six months of life (1). It is considered an essential source of nutrients containing water (87%), fat (3.8%), proteins (1.0%), and lactose (7%), with both lactose and fat providing 40 and 50% of the total energy received from milk (2). BM also contains immune cells, microRNAs, hormones and bioactive compounds with anti-inflammatory, anti-infective properties (3). These include cytokines, chemokines, immunoglobulins, hormones, growth factors, oligosaccharides and antimicrobial peptides such as bacteriocin and lactoferrin (4). Studies have shown that the composition of BM varies depending on maternal and environmental factors, and is tailored to the baby's complex nutritional requirements (5).

Our understanding of the origin of milk microbiota and its role in seeding the infant's gut is still in its infancy and needs further research (6). The delivery mode appears to be an important factor in the development of the infant's gut microbiota (7, 8). Babies born via Caesarian section (C-section) are colonized by microbial communities similar to their mothers' skin microbiota, opposed to the vaginally-delivered babies whose microbiota is closer to their mothers' vaginal microbes (9). Furthermore, it is known that the rupture of the membranes during labor contributes to the early microbial seeding of the newborn (10). This transfer of microbes from the mother to her baby, during delivery, is like a “good starter kit” that will help expand the infant's microbiota.

Studies have shown that breastfed infants have higher levels of *Bifidobacterium species* in their gut, which has been attributed to the human milk oligosaccharides (HMOs) known to preferentially feed this bacterium (11, 12). This is in contrast to the formula-fed infants where the gut is inhabited by *Bacteroides, Firmicutes, Eubacterium* and *Veillonella* (13). When solid food is supplemented after 6 months of age and BM is no longer considered the major source of nutrients, levels of *Bifidobacterium* in the gutdrop dramatically, while levels of *Lachnospiraceace* and *Ruminoccoceae* increase (13). During this formative stage, the child's gut microbiota continues to grow in number and diversity of its microbial communities. At around 14 months old, when most children stop receiving BM, the toddler's gut microbiota enters a transitional stage during which the microbial communities of the gut are considered unstable and can be disrupted by various factors such as antibiotics use, diet and other environmental factors (14). However, as the child grows older, the microbial diversity of the gut becomes more stable and mainly dominated by Firmicutes (*Lachnospiraceae* and *Ruminococcaceae*), Bacteroidetes (*Bacteroidaceae, Prevotellaceae*, and *Rikenellaceae*), and Actinobacteria (*Bifidobacteriaceae* and *Coriobacteriaceae*) (15).

Similar to the gradual changes observed in the gut microbiota composition, the immune system of the infant goes through different phases of maturation in early life, in response to microbial exposures that primarily take place at the mucosa of the respiratory and gastrointestinal tracts (16). Babies are born with a naive immune system making it difficult to fight infectious pathogens directly after birth (17). During lactation, BM provides a source rich with IgA, anti-inflammatory factors and immunologically active cells needed to induce both tolerance to non-harmful antigens (food antigens or beneficial commensal microbes) and to develop a robust immune defense against pathogenic organisms (18).

Currently a comprehensive understanding of the diverse composition of the BM microbiota including prokaryotes (bacteria, archaea) and eukaryotes (fungi) is lacking. In this article, we aim to review the current knowledge about the BM microbiota, its composition and role in the development of the infant's immune system.

## Methods

A search of the medical literature was performed using PubMed database for articles published from database commencement until May 2021. Initial search was carried out using the general search terms: Infant, gut, microbiota, BM, breast milk, immune system, immunity, chronic diseases, vaginally delivered, C-section. Only articles published in English were included. No restrictions were placed on the study design or the type of article. References of the included articles were also reviewed for additional relevant articles.

## Results

### Human Milk Nutrients

**Figure 1:**
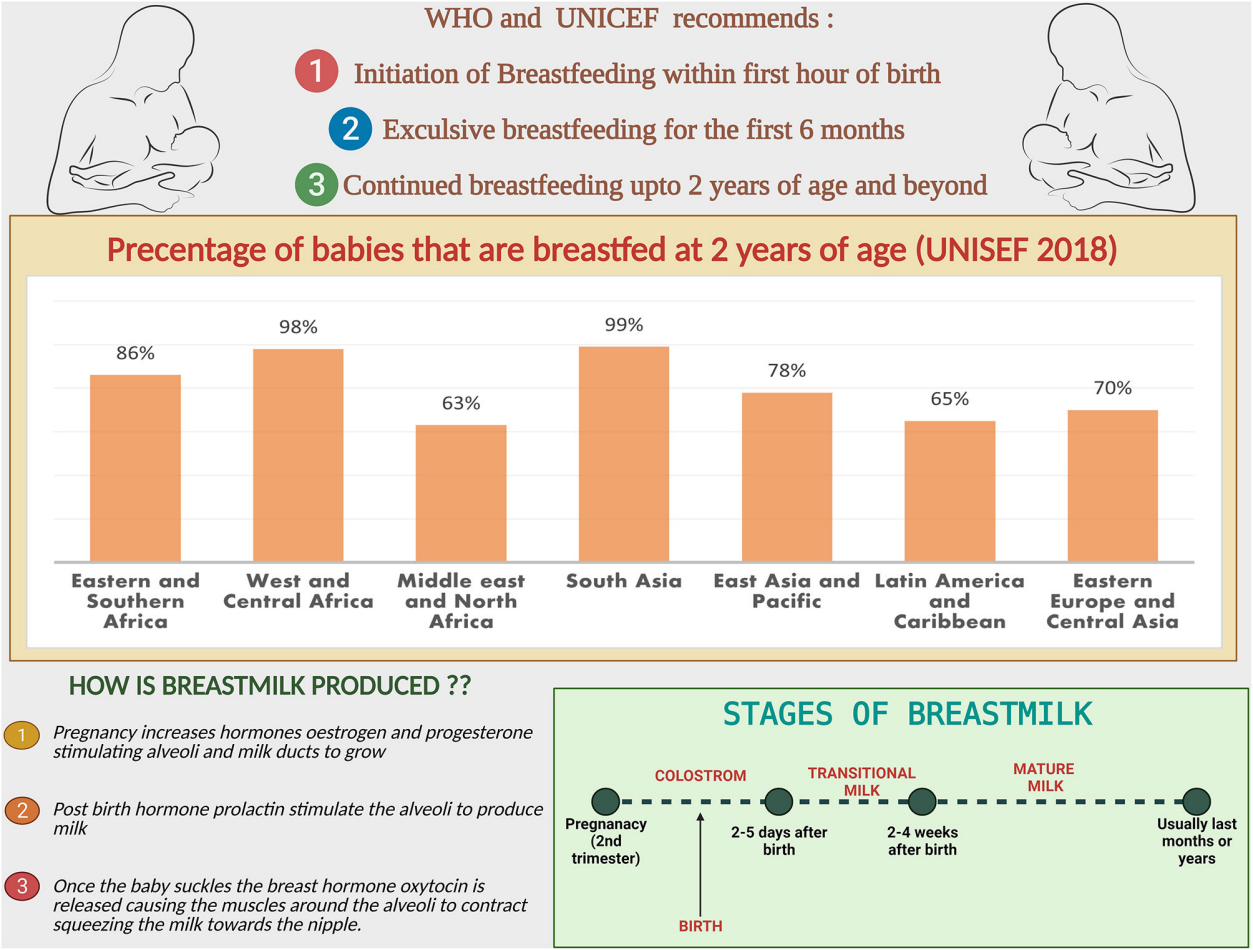
Breastfeeding facts and figures: the recommendations from WHO and UNICEF, comparison of the percentage of babies breastfed at 2 years region wise. Steps and stages of breastmilk production.

Up to 50% of infections-related death in children aged 6–23 months is thought to be caused by the lack of adequate breastfeeding (19). BM contains sufficient nutrients and bioactive compounds to provide complete nutrition for the developing infant (20). The benefits of BM are numerous and superior psychologically, economically, ecologically and nutritionally (21). WHO and UNICEF recommends exclusive breastfeeding for infants for the first six months to achieve optimal growth and development (22).

followed by continued breastfeeding up to age 2 years and beyond, for prevention of childhood malnutrition and other diseases (Figure 1) (23). The nutritional components of BM are derived from three main sources, some from synthesis in the lactocytes, some from dietary origins, and the rest originate from maternal stores (24).

Lipids, proteins and carbohydrates are the significant macronutrients found in the BM (25). Macronutrients composition varies with lactation stage, possibly due to adaptation of milk composition to the increased energy demand of the growing infant (26). When compared with mature BM, pre-colostrum milk contains more minerals and protein, but less sugar and fat (27, 28). Another study looked at the macronutrient content of pre-colostrum milk and showed that it has 3.7 g of proteins, 2.9 g of fat and 5.3 g of lactose (29). In comparison to the mature BM macronutrient composition, proteins are estimated to be 0.9 to 1.2 g/dL, with fat at 3.2 to 3.6 g/dL and lactose at 6.7 to 7.8 g/dL (24).

The main source of energy in the BM is fat, and BM fatty acids are known not only as a major energy source, but also as important regulators of development, immune function, and metabolism (30, 31). It is recommended that the mother consumes a well-balanced diet to ensure adequate polyunsaturated fat transfer to human milk.

Lactose is the primary carbohydrate found in milk, accounting for 40% of the baby's energy, it is relatively constant in mature milk and aids minerals and calcium absorption (32, 33). HMOs are lactose-based unconjugated carbohydrates that are considered the third most abundant component of BM after lactose and lipids (34). Oligosaccharides are a group of complex glycans found in the milk of most mammals, but the human milk oligosaccharides profile is thought to be the most diverse with over 200 distinct HMO structures identified in the human BM thus far (35, 36). Given their inhibitory properties, HMOs play an important role in preventing the adhesion of microorganisms to the intestinal mucosa, thereby protecting against the disease-causing organisms in the baby's gastrointestinal tract (4, 37). Some HMOs exhibit antimicrobial and antibiofilm properties against *Group B Streptococcus* (GBS) (38). GBS is one of the most common pathogens responsible for neonatal infections in full-term newborn infants during the first week of life (39). Humans rely primarily on their gut bacteria, including *Bifidobacterium*, to digest HMOs due to the lack of enzymes (37, 40). The monosaccharides used as building blocks for HMOs are glucose (Glc), galactose (Gal), N-Acetyl-Glucosamine (GlcNAc), fucose (Fuc), and sialic acid (Neu5Ac) (41). The lactose HMO backbone can also be fucosylated or sialylated to form trisaccharide HMO structures, termed 2′ or 3′-fucosyllactose (2′or and 3′ or 6′-sialyllactose (3′ or 6′SL) respectively (11). HMOs modulate neonatal immunity in the infant gut by binding to the cell surface receptors expressed on epithelial and immune cells (42). HMOs have the potential to mimic viral receptors and block adherence to target cells, thus preventing infection (43).

The protein content of BM is low but highly bioavailable, and it appears to play an important role in the growth of the infant (44). Protein gain in an infant's body is greatest in the first months of life, when protein concentrations in BM are higher than in later stages of lactation (45). In the BM, there are two classes of protein including casein and whey, casein is known to form clots or curds in the stomach; whereas whey remains liquid and is easier to digest (46). Whey accounts for 50 to 80 percent of the protein content in BM, depending on the stage of production (24). There are over 20 different amino acids found in breast milk, such as free amino acids (FAAs) (47). These amino acids play a role in infant immune development. Glutamate and glutamine are major component of the FAAs and support growth of the nervous tissue and intestines (48) followed by Taurine that combines with bile acids to aid in the development of the brain and the eye (48). Additionally BM contain an array of native proteases and protease inhibitors such as carboxypeptidase B2, plasmin, kallikrein, elastase, thrombin, cathepsin D etc. most of which are active and lead to hydrolysis of the milk proteins to release peptides that are relevant to the developing infant (49, 50).

Lactoferrin (Lf) is a multifunctional protein that is found in high concentrations in human milk (51). Lf is an iron-binding glycoprotein that has been linked to a variety of biological functions, including the promotion of cellular proliferation and differentiation, as well as antimicrobial, anti-inflammatory, immunomodulatory, and prebiotic properties (51). Various factors may affect its concentration in BM, such as the stage of lactation, ethnicity, and diet (51). Lactoferrin concentration in BM range from 7 mg/ml in colostrum to 1 mg/ml in mature milk (52). LF binds free iron which is an essential nutrient for the growth of beneficial bacteria such as *Lactobacillus* and *Bifidobacterium* (53). This protein has antimicrobial, anti-inflammatory, and immunomodulatory properties that help to maintain homeostasis and control life-threatening diseases in the intestine of neonates (54). Milk is an enriched source of Lf and it also contains lactoferricin (Lfcin), a peptide derived from the Lf N-terminus after gastric digestion that has antimicrobial activity against pathogens (55). Lf and Lfcin inhibit the growth of gut-beneficial microorganisms, but interestingly, both Lf and Lfcin promote the growth of specific probiotic strains such as *bifidobacteria* and *lactobacilli* (56). Summary of the main immunological componenets of breastmilk and thei functions have been listed in Table 1.

**Table 1:** Immunological components of BM along with their function.

**Immunological component of BM**	**Function**
Maternal antibodies	IgA, IgM, IgG and secretory versions of IgM (SIgM) and IgA (SIgA) primarily bind to the microbes, protecting the respiratory and gastrointestinal tracts (57)
Human milk oligosaccharides (HMOs)	Selectively stimulating the growth of beneficial bacteria in the intestine thus acting as prebiotic agents while inhibiting pathogens from adhering to their target receptors in the host gastrointestinal tract (58)
Lactoferrin	Iron binding glyocproteins with anti-bacterial activity (59)
Cytokines and growth factors	Acts as signals from the mother to her infant, and enhance the anti-infective function of leukocytes (60)
Defensins and cathelicidin	breast milk contains high concentrations of multiple defensin and cathelicidin that act as antimicrobial peptides (60)
Lysozyme	Have enzymatic activity that cleaves the cell wall and the outer membrane of microbes causing lysis (60)
Leukocytes	Includes lymphocytes, macrophages and neutrophils with primary role to protect the mammary gland against infections (57)
Free aminoacids and proteases	support growth of the nervous tissue, eye and intestines and proteases helps in the hydrolysis of the milk proteins (47, 49)

**Figure 2:**
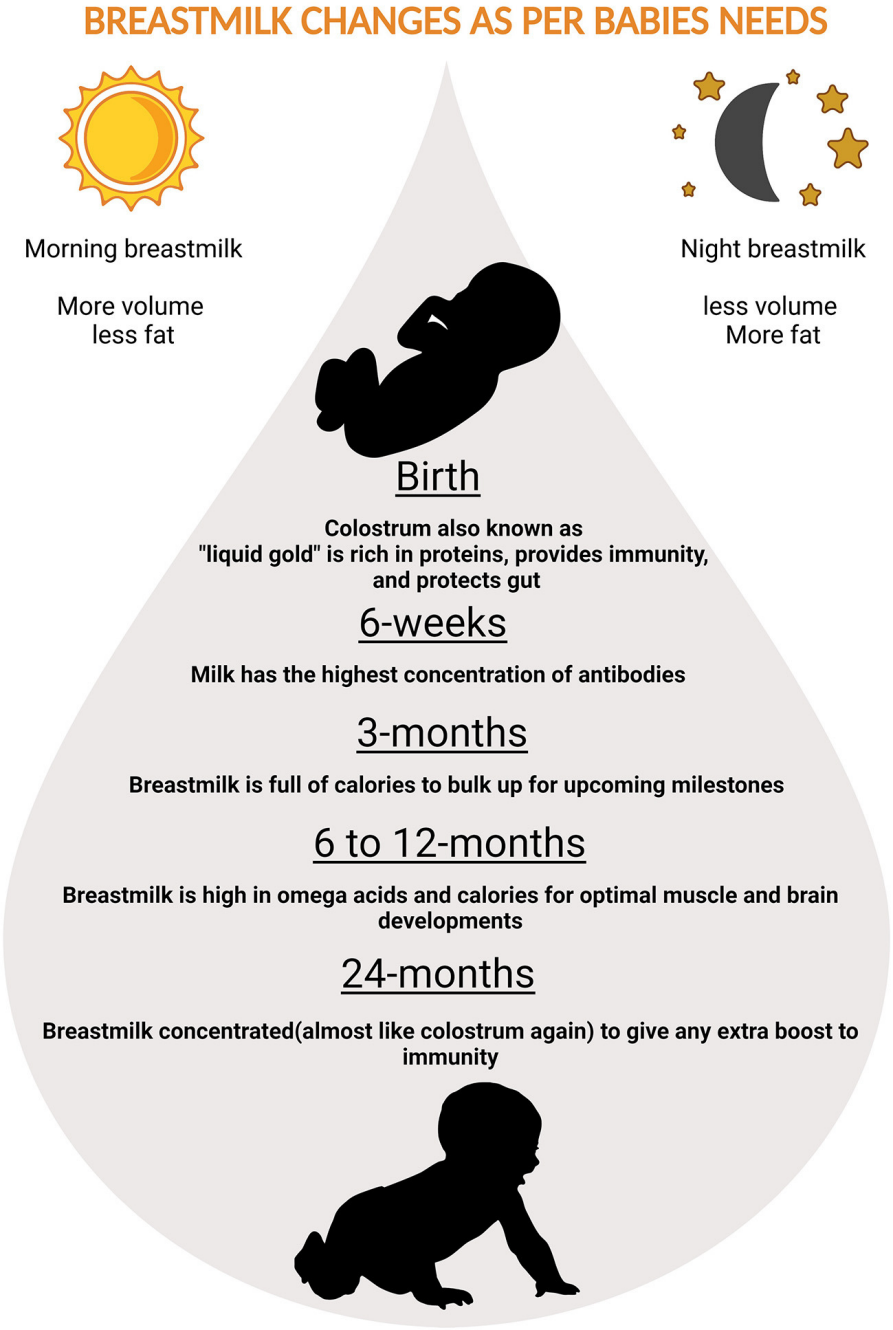
Magical ways in which breastmilk changes to adapt to babies needs.

Nutrients in the BM adapt to needs of the growing baby. In the first few days thick, honey-textured colostrum also called as the “liquid gold” is packed with immunological components that protect the newborn and serves as “baby's first vaccination” (61). The secretory immunoglobulin A (SIgA), present in colostrum coats the internal organs and lining of the digestive tracts preventing it from pathogens. After supercharging the baby's immune-system in the first two or three days, BM increases in volume and changes to transitional milk lasting roughly three to seven days, characyerized by higher levels of proteins, fat as well as increased lactose to provide energy (61). By the end of the first two weeks, mature milk begins to appear bluish is thinner and watery (90% of it is water necessary to keep the infant hydrated), 10% is comprised of carbohydrates, proteins, and fats which are necessary for both growth and energy (61) (Figure 2). It is also worth noting that BM content changes in term and preterm deliveries (62). Preterm BM contains significantly higher concentrations of protein, sodium, chloride, magnesium and iron (63), in addition to higher levels of fat (64–66).

### The Breast Milk Microbiota

While initially considered a sterile fluid, several studies concluded that BM is home to diverse microbes known as the human milk microbiome (HMM) (57, 59, 60). According to the Developmental Origins of Health and Disease (DOHaD) hypothesis, early-life environmental exposures can alter fetal and infant programming, resulting in changes in health status (67). Early life microbiota is recognized as a key participant in the DOHaD hypothesis contributing to infant health status in the short and long term (67). Due to the evolving scientific interest in the benefits of the HMM, it is critical to investigate its origin and detailed composition.

### Origin and Sources of the Human Milk Microbiota

BM is a source of microbes necessary for the establishment of the oral and gut microbiota in breastfed infants (68). Many hypotheses have been proposed to explain the origin of these microbes in BM, Fernandez et al. proposed “retrograde transfer” of external bacteria and the “entero-mammary pathway” for internal bacterial translocation (69). Recent research has also suggested “oro-mammary translocation” (70, 71).

External bacteria could enter the mammary gland via “retrograde transfer” from sources such as areola skin, infant oral cavity, and/or breast pumps. The reverse flow of milk back into the breast during breastfeeding and pumping may allow microbes to enter and establish in the milk ducts from the infant's mouth (69). The fact that women who use a breast pump and have cracked and/or sore nipples are more likely to develop lactational mastitis (72) supports the retrograde transfer of exogenous microbes.

The “entero-mammary pathway,” on the other hand, occurs when bacteria from the mother's gastrointestinal tract are translocated to the mammary glands via immune cells and colonize the available niche (69). Altered tight junction in the intestinal tract, particularly in the late stages of pregnancy, can predispose to bacterial translocation (73). Vertical transmission of microbes such as bacteria and viruses through breastfeeding (74), the presence of a distinct colostrum microbial community before the start of infant feeding (75), and the isolation of orally administered probiotic strains such as *lactobacilli* from the BM (76) all support the entero-mammary pathway. A recent study with a single mother-infant pair found intriguing evidence for microbial exchange via the entero-mammary pathway, with the same strain of *Bifidobacterium breve* found in the mother's gut, her BM, and in the infant's gut (6). This infant was delivered via C-section limiting the possibility of maternal microbe colonization during delivery (6). The authors showed that, while *Bifidobacterium breve* constituted less than 1% of the maternal rectal sample, it made up to 28% of the BM microbial composition and 68% of the infant's gut microbiota (6). Similarly, comparison of BM and infant stool microbiota in 10 mother–infant pairs from birth to three months of age found 12 predominating 12 core genera: *Pseudomonas, Staphylococcus, Streptococcus, Elizabethkingia, Variovorax, Bifidobacterium, Flavobacterium, Lactobacillus, Stenotrophomonas, Brevundimonas, Chryseobacterium*, and *Enterobacter*. The fact that the genera shared by infant feces and human milk samples accounted for 70–88 percent of the total relative abundance in infant fecal samples lends support to vertical transfer of bacteria from BM to human gut (77). Finally, it is possible that microbes in the mother's oral cavity will also translocate to the mammary gland. Several studies have found similarities between maternal oral and milk microbiota, supporting the “oro-mammary translocation” pathway. More research is needed to confirm the extent to which each of the mechanisms described above influences the microbiota load and composition of milk (78).

### Composition

Culture-dependent techniques have traditionally been used to confirm the presence of microbes in BM. Next-generation sequencing and omics technology advancements (79) has opened up new avenues for studying the HMM from microbiological and immunological perspectives. This section summarizes the main advances on our understanding of the HMM composition made possible by traditional and refined tools, as well as opportunities to close existing knowledge gaps.

#### Bacteriome

Exploration of milk microbiota has been made possible through the use of both culture-dependent and culture-independent methods. Culture-dependent methods only target cultivable bacteria, and isolates are highly dependent on the media used, sample storage conditions, and growth conditions. On the other hand, molecular techniques such as denaturing/temperature gradient gel electrophoresis (DGGE), single-strand conformation polymorphism (SSCP), restriction fragment length polymorphism (RFLP), and quantitative polymerase chain reaction (q-PCR) rely on direct extraction of bacterial DNA. High-throughput 16S targeted sequencing or whole genome metagenomics detect DNA from all bacteria in a sample.

*Streptococci* were isolated from human milk in early 1924 (80) and it was thought that they were colonized through the infant's mouth during suckling (81, 82). McCarthy *et al*. found *S. salivarius* to be a common mouth isolate in infants (82). Numerous studies on the microbiology and biochemistry of BM revealed the presence of rapidly growing culturable microorganisms, including Gram-positive species (*Staphylococcus, Streptococcus, Corynebacterium*, and *Propionibacterium*) (83, 84). Carroll et al. performed aerobic cultures of 207 samples of drip breast milk collected from 70 mothers, and showed that 82% contained only *Staphylococci* and *Streptococcus viridans*, while 17% grew potential pathogens such as *Staphylococcus aureus, Enterobacteria* or group B *Streptococci* (85). Other bacterial groups found in BM may require specific conditions or specialized media. For example, *Lactobacillus, Lactococcusm* and *Bifidobacteria* have only been isolated from milk after using specific growth media and anaerobic incubations (86–89). It has been reported that over 590 different genera and 1300 bacterial species have been found in BM using various techniques to date (3), but the number of cultivable bacterial species in a given individual at a given time point ranges from 2 to 18 (90).

Noncultural analysis of BM began in the early 2000s with the use of DEGG and 16S rRNA clone library analysis, as well as q-PCR, which confirmed the general composition described by culture studies while highlighting a much broader array of microorganism diversity. These methods have revealed a dominance of *Staphylococci, Streptococci, Propionibacteria* and *Bifidobacteria*. Moreover, DNA from other bacterial groups, such as *Weissella, Clostridium* and *Serratia*, was also detected (91, 92).

Hunt et al. performed the first deep sequencing analysis on breast milk collected from 16 healthy women (20–40 years of age) nursing infants (93) revealing *Streptococcus, Staphylococcus, Serratia, and Corynebacterium* as the most abundant genera. The use of next generation sequencing technology has revealed an even more diverse bacterial population, as well as a better understanding of the factors that may influence its composition. A study that used a combination of culture-dependent and culture-independent methods (Sanger sequencing and 454-pyrosequencing) to examine the microbiota of BM from seven lactating women at three different time points, and found *Staphylococcus, Streptococcus, Bifidobacterium, Balutia, Brevundimonas, Corynebacterium, Flavobacterium, Propionibacterium, Pseudomonas, Ralstonia, Rothia*, and *Burkholderia* as the most abundant genera (94). Chen et al. discovered five genera of microbiota in milk samples collected from 33 women: *Staphylococcus, Streptococcus, Enhydrobacter, Enterococcus*, and *Rothia* (95). Despite the fact that the majority of the above studies sought to identify the BM microbiota of healthy women following birth, differences in the core genera between these studies are evident. Shotgun metagenomics and 454 pyrosequencing have also been used in studies to compare the metagenomes of human milk samples provided by healthy and mastitis-suffering women (96). The healthy core microbiota included the genera *Staphylococcus, Streptococcus, Bacteroides, Faecalibacterium, Ruminococcus, Lactobacillus*, and *Propionibacterium* (96). On the other hand, *Staphylococcus aureus* and *Staphylococcus epidermidis* dominated the microbiota in the samples collected from the women with acute and subacute mastitis respectively (96). Fungal and protozoa-related reads, as well as archaea and viral related reads, were also detected (96). It is also worth noting that there are significant inter-individual, inter-population, and inter-study variations, thus standardization of samples collection, processing and analysis is required. Table 2 summarizes the list of bacterial species identified in BM using various techniques.

**Table 2:** Bacterial populations detected in raw human milk using culture-dependent, culture-independent, and next-generation DNA sequencing methods.

**Culture dependent**	**Culture-independent**	**Next generation sequencing**
**Identified bacteria**		
*Bifidobacterium species (77, 92, 97–100)/adolescentis (92, 100)/bifidum (92, 99)/breve (92, 97, 99, 100)/longum (92, 97, 100)/infantis (100) pseudocatenulatum (92, 100)/dentium (99, 100)/angulatum (100)*	*Bifidobacterium species (58, 92, 97, 101, 102)/longum (92, 97)/lactis (97)/animalis (101) adolescentis (92, 101)/bifidum (92, 101)/breve (92, 97, 101)/catenulatum (101)/dentium (92)*	*Bifidobacterium (6, 77)*
*Lactobacillus species (77, 97, 99, 100, 103)/acidophilus (99)/fermentum (97, 99)/lactis (103)/mesenteroides (103) plantarum (97, 99)/gasseri (97)/crispatus (103)/rhamnosus (97, 103)/fructivorans (99)/salivarius (97)/reuteri (97)/casei (97)/gastricus (97)/vaginalis (97)/brevis (99)/helveticus (104)/oris (104)/delbrueckii (99)*	*Lactobacillus species (58, 97, 102)/fermentum (97)/casei (97)/gasseri (97)/plantarum (97)/rhamnosus (97)/reuteri (97)*	*Lactobacillus* *(77, 98, 105)*
*Lactococcus species (103)/lactis (103)*	*Lactococcus species (104)/lactis (104)*	*Lactococcus (106)*
*Leuconostoc species (103)/mesenteroides (103)*	*Leuconostoc species (104)/citreum (104)/fallax (104)*	*Leuconostoc (106)*
*Corynebacterium species (107)*	*Corynebacterium species (107)*	*Corynebacterium* *(98, 108)*
*Enterococcus species (100, 103)/faecium (104)/faecalis (103, 104)/durans (104)/hirae (104)/mundtii (104)*	*Enterococcus (58, 103, 107) faecalis (103)/faecium (104)*	*Enterococcus (106, 108)*
*Streptococcus species (97, 100, 103)/mitis (97)/salivarius (97, 103)/oris (104)/parasanguinis (97)/peroris (103)/oralis (103)/lactarius (104)/australis (104)/gallolyticus (104)/vestibularis (104)*	*Streptococcus species (58, 102, 103, 107)/mitis (103)/parasanguis (104)/salivarius (103)*	*Streptococcus (77, 98, 105)*
*Staphylococcus species (97, 100, 103, 109)/epidermidis (97, 100, 103)/aureus (100, 103)/capitis (103)/hominis (103)/lugdunensis (103)*	*Staphylococcus species (58, 102, 103, 107)/epidermidis (103)/hominis (103)*	*Staphylococcus (77, 98, 106)*
*Rothia species (107)/mucilaginosa (107)/dentocariosa (107)*	*Rothia (102, 107) Clostridium species (58)*	*Rothia (98)*
*Weissella (110)*	*Weissella species (104)/cibaria (104)/confusa (104)*	*Weissella (106)*
*Propionibacterium (69)*	*Propionibacterium species (111)/acnes (111)*	*Propionibacterium or Cutibacterium (98, 105, 108)*
*Acinetobacter (98, 100, 109)*	*Acinetobacter (107)*	*Acinetobacter (98, 106, 108, 112)*
*Pseudomonas (109)*	*Pseudomonas (107)*	*Pseudomonas (77, 105)*
*Kocuria species (98)*		*Kocuria (105)*
*Escherichia (100, 109)*		*Escherichia/Shigella (112, 113)*
	*Akkermansia (58)*	*Akkermansia (106)*
	*Enhydrobacter (107)*	*Enhydrobacter (98)*
*Actinomyces (98)*		*Actinomyces (105)*
*Citrobacter (100)*		*Citrobacter (106)*
*Klebsiella (100, 109)*		*Klebsiella (108)/*
*Bacillus (109)*		*Bacillus (108)*
*Gemella (114)*	*Gemella (102)*	*Gemella (98)*
*Enterobacter (100)*	*Enterobacter (107)*	*Enterobacter (77)*
*Micrococcus (109)*	*Micrococcus (107)*	
	*Prevotella (58)/*	*Prevotella (106, 108)*
*Burkholderia (100)/Kluyvera (115)/Pediococcus species (104)/pentosaceus (104)*	*Moraxella (107)/Bacteroides (58)*	*Variovorax (77)/Pantoea (108)/Serratia (108)/Ralstonia (108)/* *Paenibacillus (108)/Flavobacterium (77)/Brevundimonas (77)/Carnobacterium (106)/Elizabethkingia (77)/Chryseobacterium (77)//Propionibacterium (98)/Aeromonas (108)/Ruminococcus (108)/Clostridium (108)* *Sphingomonas (106)/Sphingobium (105)/Ottowia (105)/Veillonella (98)/Bradyrhizobium (113)/Granulicatella (116)/*

BM can also serve as a vehicle for pathogenic bacteria, several outbreaks and case reports of neonatal diseases such as Group B *Streptococcus* (GBS) (117–120), *Bacillus cerus* (121, 122) *Staphylococcus aureus* (MRSA) (123), have been related to tainted breast milk (74).

GBS is a leading cause of neonatal sepsis and meningitis in developed countries (124) with two distinct syndromes: early-onset disease and late-onset disease (125). Several studies have found a link between GBS colonization in breast milk and late-onset sepsis (LOS), but the causality of the link is unclear (126). Heavy colonization and recurrent infection have been observed in newborns whose GBS late-onset sickness is presumed to be caused by breast milk (127). A total of 59 cases have been identified of infants in which contaminated BM was associated with GBS LOD out of these infants 49% were term and 51% preterm (127). GBS strains were detected in 30 infant-mother pairs in the above cases, where the infant's strain was identical to that found in the mother's breast milk (127). In this review we had earlier discussed different possible mechanisum of the origin of breast milk microbiome via retrograde transmission from the infant: similarly GBS colonization of the infant during delivery or after birth can lead to contamination of the maternal mammary ducts, GBS can then multiply in the mammary ducts, resulting in an increase in bacterial concentration in the milk, which re-exposes infants during breastfeeding, resulting in persistent exposure/colonization of both the infant and the mother (117, 128, 129). Another route proposed for GBS contamination of breast milk is transfer from the maternal gastrointestinal system to the mammary glands via lymphatics (129). Septicemia, respiratory tract infection, enterocolitis, hepatitis, endocarditis, endophthalmitis, and encephalitis with brain abscess are other severe diseases that *B. cereus* can cause, especially in children (130). In two very low birth weight neonates, a cluster of severe intestinal infections due to *B. cereus* was reported, with pooled breast milk suspected as a source of contamination (131) and BM was also suspected as a possible source of B. cereus infection in three premature neonates admitted to intensive care units in two hospitals in Île-de-France (132). Furthermore, the environment in which milk is expressed, collected, transported, stored, and handled could introduce B. cereus into milk. Contaminated pumps have been discovered as bacterial contamination reservoirs, particularly after being used by numerous mothers and not being cleaned properly. MRSA, on the other hand, has been linked to mastitis (breast infection) and breast abscesses in breastfeeding moms, and it can also be transferred from mother to preterm newborn via contaminated breast milk, even if the mother is not infected (123). Thus adoption of more strict measures such as appropriate handling and bacteriological screening of milk to identify possible pathogens is crutial to control transmission of infection.

#### Virome

**Figure 3:**
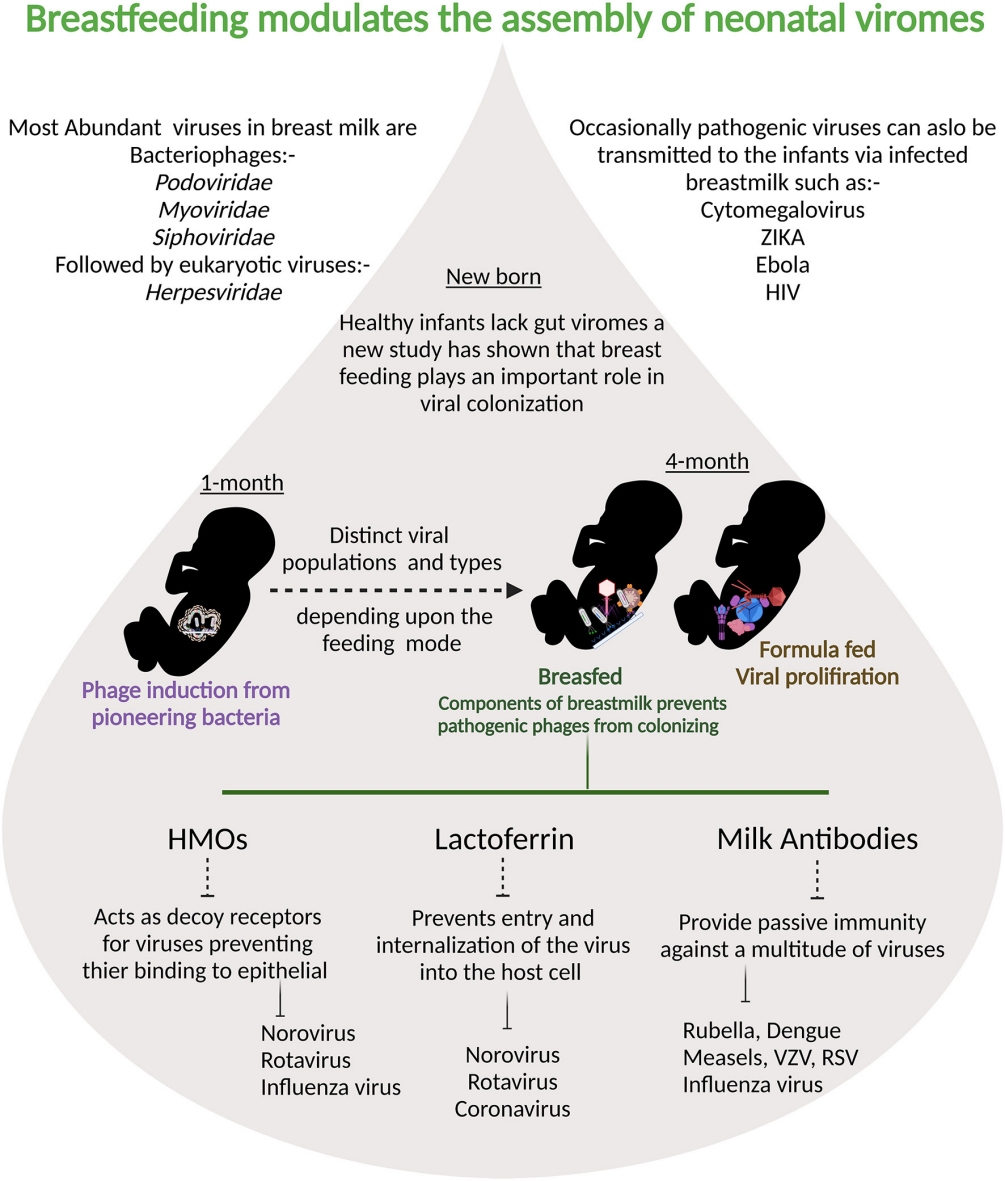
Breastmilk ensures that fewer pathogenic viruses colonize infant intestine: at one month prophages are induced from the pioneering bacteria providing the first population of virus-like particles. By four months of life, multiple human viruses are abundantly detected in stool samples from babies.Viral populations differ depending on the feeding mode and components of breast milk are protective against viral infections.

Human milk viruses, including eukaryotic viruses, bacterium-infecting viruses known as bacteriophages, and other viral particles, have been found to be transmitted from mother to infant via breastfeeding (133). Human breastmilk milk viruses play an important role in shaping the infant gut virome and microbiota because bacteriophages can kill bacteria or provide them with potentially beneficial gene functions (133). Breastmilk modulates and supports stepwise assembly of baby viromes beginning at one month, according to a recent study, and breastfeeding was linked to less human viruses in infants' guts than formula-feeding alone (134). Components of breastmilk such as HMOs, lactoferrin, and maternal antibodies also negatively influence the internalization of pathogenic viruses (Figure 3). The majority of the viruses detected in breast milk were bacteriophages from the Myoviridae, Siphoviridae, and Podoviridae families, according to a study of healthy women in the United States (135). Bacteriophages have been the most extensively studied component of the human virome to date, they account for the vast majority (95 percent) of viruses found in human milk and infant stool (135). Evidence suggest that the mother-to-infant virome transmission occurs, as BM and stool viromes from mother-infant pairs shared a significant homology of bacteriophages (135, 136). Recent research on the role of immunosuppression on the bacteriome and virome of breast milk in HIV-positive women discovered that bacterial and viral communities are resilient in breast milk despite immunosuppression (137). There is a definite need for more longitudinal paired mother- infant studies designed to capture the dynamic nature of the milk and infant virome. Additionally, studies are needed to examine effects of breastfeeding duration, maternal health, age, geographic variation, and other factors on the milk and infant virome. Although virus transmission through breast milk is uncommon, viral pathogens such as hepatitis B virus (HBV), hepatitis C virus (HCV), cytomegalovirus (CMV), West Nile virus, human T-cell lymphotropic virus (HTLV), and HIV have been found in breast milk (74). CMV is the most prevalent congenital infection in the United States (138) and it is found in the breast milk of CMV-positive women at rates ranging from 13 to 50% (139–141). Mothers who were CMV-seronegative did not shed virus in their BM. CMV transmission was only identified in infants of seropositive women who excreted CMV and breast-fed their children, according to Vochem et al. (139). For the first time, potential West Nile virus transmission via human milk was reported in September 2002. The Centers for Disease Control and Prevention has been collecting reports of West Nile virus infection in mothers or infants during the nursing period since 2003 (142). Similarly, possible Zika virus transmission through human lactation was investigated, and the World Health Organization concluded that “the benefits of breastfeeding for the infant and mother outweigh any potential risk of Zika virus transmission through breast milk (143).” HBsAg transmission in breast milk of chronically infected mothers was confirmed in 1970 (144). Other studies later discovered not just HBsAg in breast milk, but also HBeAg and HBV DNA. Furthermore, colostral HBsAg and HBeAg titers are positively correlated with the corresponding amount in maternal blood (145). The World Health Organization, on the other hand, believes that a mother's chronic Hepatitis virus infection is not a reason to stop breastfeeding. When a woman is exposed to infectious diseases, pathogenic agents can be transmitted through human milk, and the healthcare professional must make an informed decision about whether or not to stop breastfeeding in each case.

#### Mycobiome

Fungi are an important but often overlooked component of the human microbiota (146). Despite the fact that fungal and bacterial colonization occur concurrently during early life (147), most infant microbiota studies have overlooked fungi. Only few studies have have assessed and confirmed the presence of potentially viable fungi in human milk (96, 148–151). According to one metagenomics study of human milk, fungi make up 0.5–2% of the milk microbial community (96). The presence of a variety of fungal species in BM has been investigated using high-throughput sequencing, microscopy and other culture-independent techniques, which revealed the presence of *Malassezia, Candida* and *Saccharomyces* as the most common genera (148). In a subsequent study, the authors analyzed BM samples from healthy adult women in South Africa, Finland, China, and Spain with normal weight (20 women per country) to investigate the potential influence of geographical location and mode of delivery on the presence of mycobiota and its composition. They discovered that Malassezia and Davidiella were the most abundant genera across the four countries, regardless of geographical area or mode of delivery (149). Furthermore, BM samples from all participants shared a core mycobiota consisting of *Malassezia, Davidiella, Sistotrema*, and *Penicillium* (149). Another study of mother-infant dyads discovered that maternal age, blood type, antibiotics, vaginal delivery, and infant gender were all linked to Candida colonization of the infant, and in a subset, maternal vaginal and rectal samples were identified as potential sources of this taxon (152).

#### Archeome

Because of advances in sequencing technologies, the human archaeome has recently gained a foothold in microbiota research (153). Methanogenic archaea *Methanobrevibacter smithii*, is thought to be the main component of human-archaeal-bacterial mutualism, in which it improves energy harvest by consuming end products of microbial fermentation via methanogenesis (154, 155). Vertical transmission of methanogenic Archaea for metabolic phenotype inheritance is a possibility. Until recently, cultivating methanogenic archaea was a laborious, expensive, and time-consuming process. Recent research, however, has used a new antioxidant-based culture system, as well as genome sequencing, to investigate the presence of methanogenic archaea in human colostrum and milk (156). Interestingly, the study found methanogenic archaea in colostrum and human milk. Two species of *Methanobrevibacter* namely, *M*. *smithii* and *M*. *oralis*, have been identified by culture and confirmed by genome sequencing (156). These findings pave the way for future research into the mechanisms underlying the transmission of methanogenic archaea as critical commensals to infants via breastfeeding.

### Factors Influencing the Composition of Human Milk Microbiota

BM contains the nutritional and immunological elements required for the development of the infant. Aside from these elements, this fluid contains a community of microorganisms known as the microbiota, which is dominated by Staphylococcus, Streptococcus, Lactobacillus, Pseudomonas, Bifidobacterium, Corynebacterium, and Enterococcus (3). The BM microbiota, like many other components, is highly dynamic, and its composition is influenced by both intrinsic and extrinsic factors such as the stage of lactation, the mother's diet, and others.

### Stage of Lactation

The composition of the BM microbiota may change during the various stages of lactation. Initially, the bacterial composition of colostrum is more abundant, particularly for the lactic acid bacteria *Weisella* and *Leuconostoc* (106) whereas the abundance of *Bifidobacterium* though low in colostrum increses in the transient milk (106). Several studies suggest that some of these bacteria are essential for the colonization of the infant's gut and the subsequent establishment of its microbiota, which prevents infectious diseases and aids in the maturation of the immune system (3). After ten days, the relative abundance of *Methylobacterium, Rothia, and Granulicatella* in BM increases (157). In the following months, the lactic acid bacteria remain as the most abundant; however, other taxa suffer variations. Between the first and sixth month, the oropharynx and gut-associated bacteria, *Veillonella, Leptotrichia, Prevotella, and Pseudomonas* dominate the BM (106).

### Method of BM Expression

The methods of BM expression, whether direct from the breast or indirect from other sources, influence the microbial composition of the BM. Direct breastfeeding is associated with a higher abundance of oral cavity-associated genera *Gemellaceae, Vogesella*, and *Nocardioides* (68). In contrast, indirect breastfeeding is associated with increased prevalence of potential opportunistic pathogenic families such as Enterobacteriaceae, Stenotrophomonas, and Pseudomonadaceae, while levels of *Bifidobacterium* decrease (68). Another study found a higher abundance of *Bifidobacterium, Ralstonia*, and *Lactobacillus* and a lower prevalence of *Staphylococcus* and *Escherichia/Shigella* in women's milk obtained by direct breastfeeding (158). Overall, bacterial diversity and richness decrease, regardless of the milk expression method. In conclusion, direct breastfeeding might allow the acquisition of oral associated microorganisms, whereas indirect breastfeeding could favor the colonization with environment-related microorganisms that could compromise the infant's immune system and increase the risk of some diseases (68).

### Lifestyle and Diet of the Mother

Lifestyle factors, particularly diet and antibiotic use, are thought to be important in modulating the composition of the human microbiota. When taken during labor, it has been shown to increase the microbial alpha diversity and richness in BM while decreasing the presence of *Bifidobacterium* and *Lactobacillus* (3, 106). On other hand women receiving anti-cancer chemotherapy during breastfeeding showed reduction in *Bifidobacterium, Eubacterium, Staphylococcus and Cloacibacterium* (159). Furthermore, diet and antibiotic consumption may vary by geographical location, resulting in differences in breast milk microbiota profiles (93, 160).

Women in the United States have lower levels of *Lactobacillus* and *Bifidobacterium* than women in Europe (93). Instead, Spanish women have a higher abundance of *Bacteroidetes, Propionibacterium*, and *Pseudomonas*, whereas Finnish women have higher levels of Firmicutes and lower levels of Proteobacteria (160). Meanwhile, samples collected from South African and Chinese women have higher levels of *Proteobacteria*, and *Actinobacteria* respectively (160). Some studies show that the mother's diet has an effect on the BM microbiota via the entero-mammary pathway (6). During pregnancy and lactation, bacteria from the maternal gastrointestinal tract translocate into the mammary gland, altering the mammary and BM microbiota (6). Thus, as the ingestion of certain nutrients influences the gut microbiota composition, this could also indirectly impact the microbial composition of the BM microbiota (78). A higher intake of fatty acids correlates with a positive relative abundance of Proteobacteria and with a negative relative abundance of *Corynebacterium* (157). Additionally, the consumption of polyunsaturated/linoleic fatty acids is linked to *Bifidobacterium* (161). Total carbohydrate and lactose intake inversely correlate with the relative abundance of Firmicutes (157). Certain vitamins and minerals also have a significant impact on the BM microbiota. Vitamin C consumption is associated with the presence of Staphylococcus, whereas thiamine, riboflavin, and folate consumption is associated with the presence of *Enterococcus* (161). Riboflavin and calcium intake are positively related to *Veillonella* relative abundance, whereas thiamine, niacin, folate, vitamin B-6, and chromium intake is negatively related to Veillonella relative abundance (157). Probiotic use during pregnancy and lactation also influences the BM microbiota, promoting higher bacterial loads of *Lactobacilli* and *Bifidobacteria*, and lower bacterial loads of *Staphylococci* (162). Prebiotics, such as fructooligosaccharides and galactooligosaccharides, on the other hand, do not cause significant changes in the richness and alpha diversity of the BM microbiota (163).

### Maternal Factors

**Figure 4:**
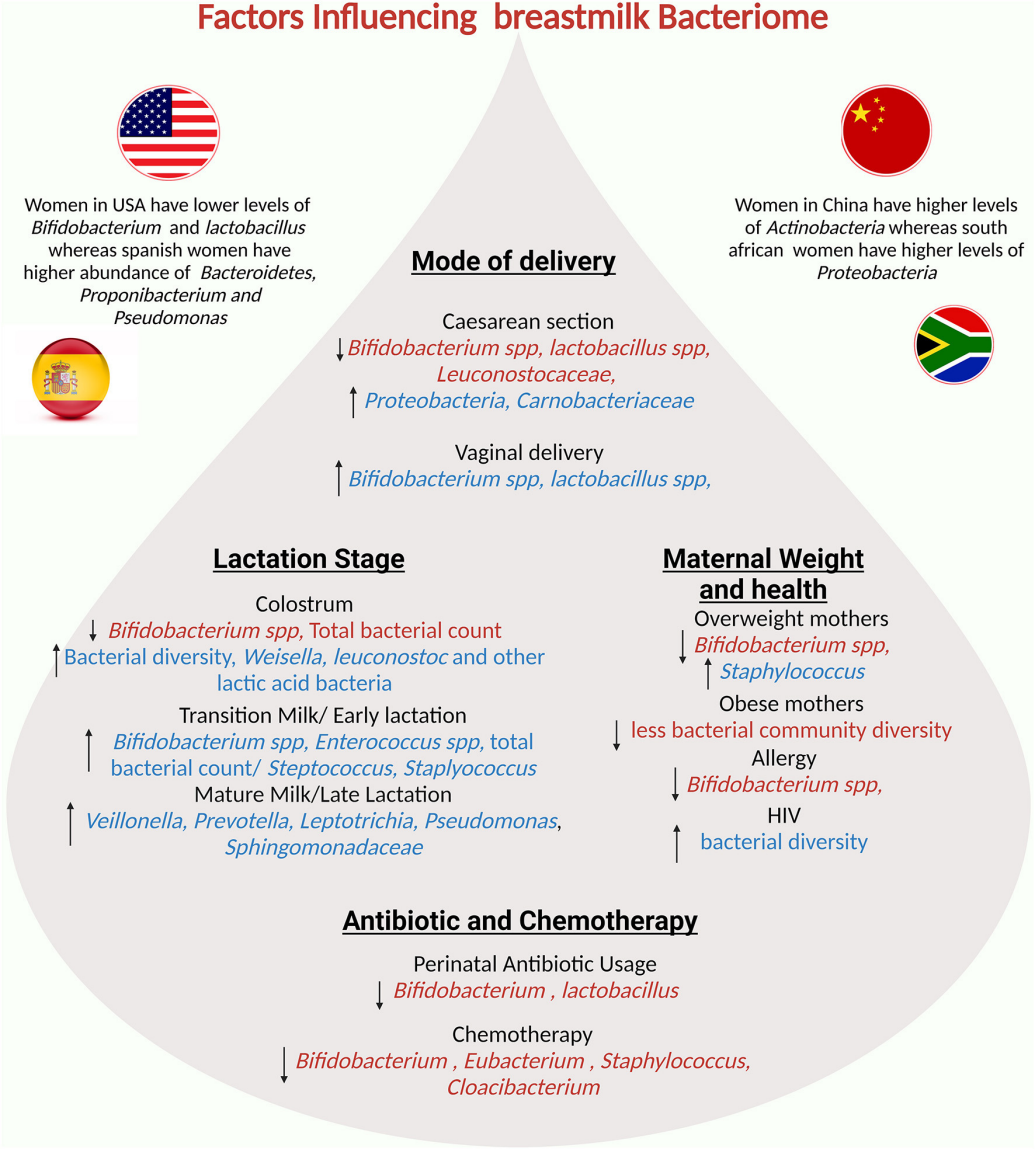
Potential factors influencing the breastmilk bacteriome: mode of delivery, maternal factors, lactation stage, and geographical region influence human milk composition.

There is a link between BM microbiota changes and women's body mass index (BMI) (106, 157). Mothers with a high BMI have a more homogeneous bacterial composition in the BM. Notably, one study discovered a correlation between having a high BMI and having a higher load of *Lactobacillus* in colostrum, a greater number of *Staphylococcus*, and a lower load of *Bifidobacterium* in mature milk (106). Another study found higher levels of the genera *Staphylococcus aureus* and lower abundance of *Bifidobacterium* in BM samples collected from overweight and obese women (106, 157). Several maternal factors are important in the microbial composition of milk. Several changes occur in the BM microbiota during lactation, particularly in the first six months. The method of milk expression is also important; indirect breastfeeding results in lower richness and diversity, as well as the acquisition of potentially opportunistic bacteria. The entero-mammary pathway shapes the microbial composition of BM based on the mother's lifestyle, geographic location, and diet. Furthermore, the body mass index influences the microbial composition of BM.Other major maternal and environmental factors influencing the composition of breastmilk are summarized in Figure 4.

### Breast Milk as an Immune Educator

The fetus' immune system is immature, and the newborn baby's immune system is considered “naïve” (164). Maternal immunoglobulin (Ig)G crosses the placental barrier to compensate and provide some protection (165, 166). The infant's immune system has limited anti-inflammatory properties after birth, and the passive immunity provided by maternal antibodies begins to fade in the first 6 to 12 months (167). Breastfed infants benefit from additional maternal protection from BM, which supplements the infant's innate immunity (57).

BM is a component of the maternal-mucosal immune system that aids in the development and regulation of both the infant's innate and adaptive immunity (168–172). BM contains various immune-regulatory components including anti-infectious agents [Lf, Lysozymes, secretory IgA (sIgA), CCl28, mucin, beta defensins, etc], anti-inflammatory agents (prostaglandins, cortisol, Interleukin (IL) 10, Tumor growth factor B1 and various antioxidants), immunomodulators 1, 2 (Il-7, IL-2, IL-18, IL-12, IL-4, IL-8 RANTES, erythropoietin, etc), and activated leukocytes (neutrophils, macrophages, and T cells) (169, 171, 172). An inverse relationship exists between the production of many proteins by the mammary gland and those in the infant (173).

Lactoferrin has been shown in animal models to have immunomodulatory properties (174, 175). Lf supplementation has been used in preterm babies to reduce the late onset of sepsis and necrotizing enterocolitis, as well as to protect against fungal infections (176). BM contains a high concentration of sIgA, which can aid the neonate in fighting potential pathogens. It has been demonstrated that sIgA binds pathogens, prevents them from contacting the intestinal epithelial layer, and traps them within the mucin layers without inducing an inflammatory response (177). Aside from sIgA, BM contains IgM, which causes pathogen agglutination, and IgG, which is known to activate phagocytosis and antigen transport to the lamina propria, resulting in B cell activation and thus promoting the infant's adaptive immunity (57).

Lactocytes, mammary stem cells, epithelial cells, and blood cells with approximately 5 × 10^6^ leukocytes are among the cells found in BM. The majority of these leukocytes are neutrophils and macrophages, which aid in the fight against microbial agents. It is now known that breast feeding for 6 months or longer reduces middle ear, lower respiratory tract infections, and allergies (24, 178–180). Toll like receptors (TLRs) such as TLR2, TLR3, TLR5, as well as soluble CD14 and human defensins, which function as pattern recognition receptors, are also found in BM (181). Furthermore, BM contains a population of highly activated switched memory cells primed to secrete antibodies (182).

HMO's absorbed during breast feeding may also play a direct role in postnatal maturation of newborn's immune system in the (183). Infants' guts contain trace amounts of sialylated oligosaccharides (184). These molecules bind to sialic acid-dependent pathogens and inhibit their adhesion to the epithelial cells of newborns and infants (185). Recent reserch has also shown that HMOS may contain tolerogenic factors influencing human DCs and thereby modulating the development of the neonatal immune system (186).

In addition to complementing innate immunity, BM contributes to adaptive immunity by shaping the thymus and promoting T-cell development (187, 188). More than 20 years ago, researchers discovered a link between breast feeding and thymic size, and another group discovered that CD8 and CD4 levels decreased in infants who stopped breastfeeding (164, 187). Another intriguing component of BM is microRNA (miRNA), a highly conserved RNA packaged in exosomes that is known to play a role in regulating immune cell development at the post-transcriptional level (189). They inhibit *in vitro* production of IL-2 and interferon-γ by stimulated T cells and increase the production of T regulatory cells (190).

### Long-Term Benefits of Breast Milk

Breastfeeding has been shown to protect against chronic non-communicable diseases (NCDs) like diabetes mellitus (DM) and cardiovascular disease (191). Breastfeeding has been shown to influence high-density lipoprotein (HDL) and cholesterol levels in adults (192). Several studies suggest that breastfeeding during the early stages of type II diabetes may improve the condition later in life, though the lack of evidence makes this difficult to conclude (193). Breastfeeding can also lower blood pressure by affecting systolic and diastolic blood pressure (194) and children who were breastfed for at least 6 months had lower blood pressure than those who were never breastfed or were breastfed for less than 6 months (192). Furthermore, epidemiological research suggest that prolonged breastfeeding reduces the risk of developing type 1 diabetes mellitus (T1DM), T1DM protection in the mouse model was attributed in part to the expansion of regulatory T cells, which may be dependent on IL-10 and transforming growth factor present in BM (195). Further breastfeeding lowers the risk of obesity allergies, asthma, eczema, Celiac Disease, Type II diabetes, among others later in the life (196).

## Conclusion

**Figure 5:**
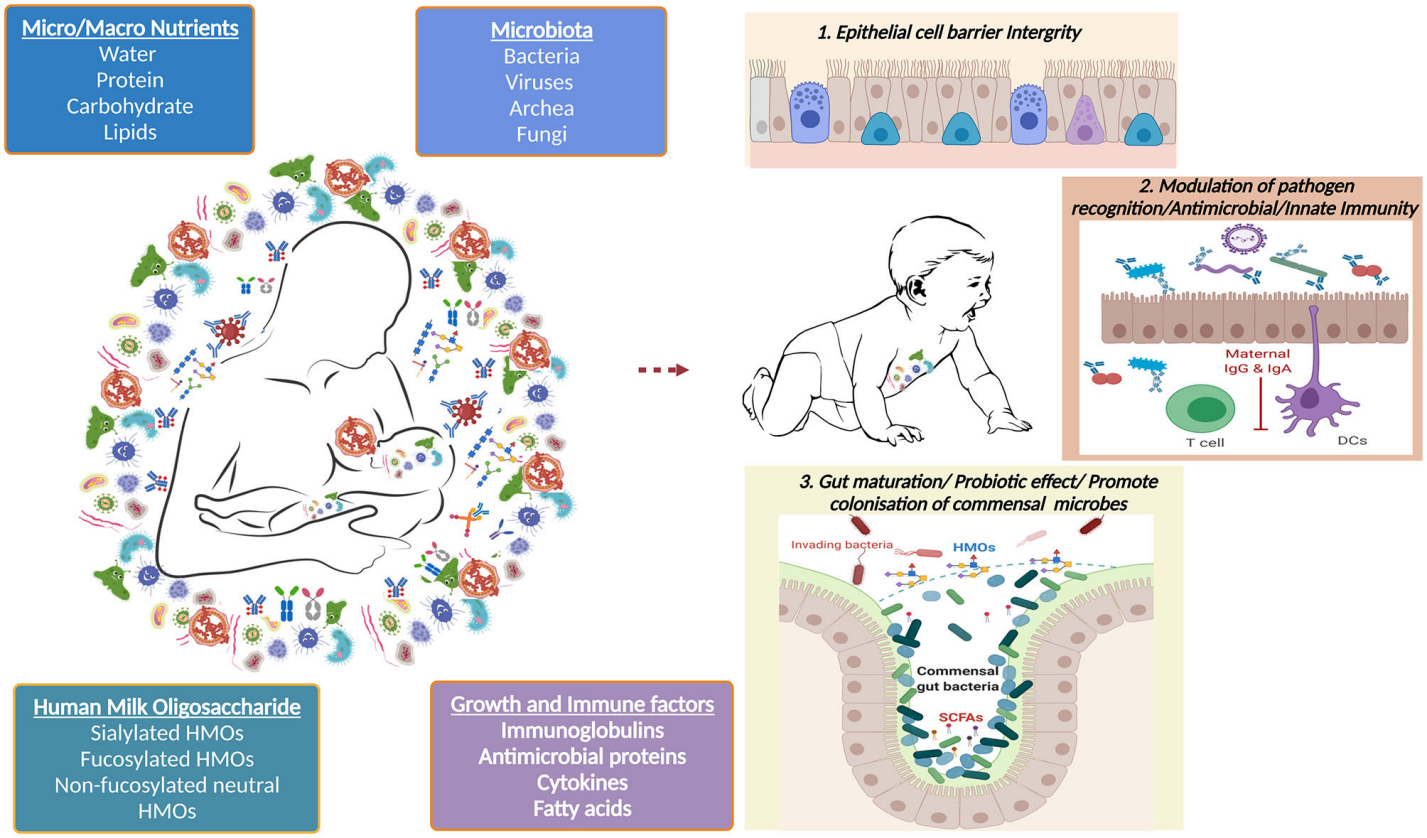
Overview of the human breast milk composition and its impact on infant's development.

The BM is a dynamic and complex microecosystem that contains a microbial signature that is transmitted to the newborn baby and is essential for immune system development and education (summarized in Figure 5). The interaction between BM, the intestinal microbiota, and the infant immune system is a developing field, and more research is needed to understand how milk immune factors promote the seeding of beneficial commensals in the gut microbiota and the development of mucosal immunity. HMOs influence mucosal immunity by promoting the growth of commensal enteric bacteria, which compete with enteric bacterial pathogens; however, this interaction may be more complex, requiring a more in-depth understanding. A complete understanding of the BM's multi-directional interaction with those players, as well as its effects on the infant, has yet to be discovered. This will entail the formation of a multidisciplinary team comprised of physicians, lactation consultants, epidemiologists, and research scientists. A multi-omics system biology approach to evaluating BM samples is the way to go.

## Author Contributions

AD and SAK: conceptualization. PS and SAK: figures and tables. All authors contributed to the article, writing, review, editing, and approved the submitted version.

## Funding

This work was supported by Sidra Medicine Internal Research Fund (No. SDR400075).

## Conflict of Interest

The authors declare that the research was conducted in the absence of any commercial or financial relationships that could be construed as a potential conflict of interest.

## Publisher's Note

All claims expressed in this article are solely those of the authors and do not necessarily represent those of their affiliated organizations, or those of the publisher, the editors and the reviewers. Any product that may be evaluated in this article, or claim that may be made by its manufacturer, is not guaranteed or endorsed by the publisher.
